# MGIT Enriched Shotgun Metagenomics for Routine Identification of Nontuberculous Mycobacteria: a Route to Personalized Health Care

**DOI:** 10.1128/jcm.01318-22

**Published:** 2023-02-22

**Authors:** Jodie A. Schildkraut, Jordy P. M. Coolen, Heleen Severin, Ellen Koenraad, Nicole Aalders, Willem J. G. Melchers, Wouter Hoefsloot, Heiman F. L. Wertheim, Jakko van Ingen

**Affiliations:** a Radboudumc Center for Infectious Diseases, Department of Medical Microbiology, Radboud University Medical Center, Nijmegen, the Netherlands; b Radboudumc Center for Infectious Diseases, Department of Pulmonary Diseases, Radboud University Medical Center, Nijmegen, the Netherlands; University of Manitoba

**Keywords:** nontuberculous mycobacteria, identification, next-generation sequencing

## Abstract

Currently, nontuberculous mycobacteria (NTM) are identified using small genomic regions, and species-level identification is often not possible. We introduce a next-generation sequencing (NGS) workflow that identifies mycobacteria to (sub)species level on the basis of the whole genome extracted from enriched shotgun metagenomic data. This technique is used to study the association between genotypes and clinical manifestations to pave the way to more personalized health care. Two sets of clinical isolates (explorative set [*n *= 212] and validation set [*n *= 235]) were included. All data were analyzed using a custom pipeline called MyCodentifier. Sequences were matched against a custom *hsp65* database (NGS-*hsp65*) and whole-genome database (NGS-WG) created based on the phylogeny presented by Tortoli et al. (E. Tortoli, T. Fedrizzi, C. J. Meehan, A. Trovato, et al., Infect Genet Evol 56:19–25, 2017, https://doi.org/10.1016/j.meegid.2017.10.013). Lastly, phylogenetic analysis was performed and correlated with clinical manifestation. In the explorative set, we observed 98.6% agreement between the line probe assay and the NGS-*hsp65* database. In the validation set, 99.1% agreement between the NGS-WG and NGS-*hsp65* databases was seen on the complex level. We identified a cluster of Mycobacterium marinum isolates not represented by the Tortoli et al. phylogeny. Phylogenetic analysis of M. avium complex isolates confirmed misclassification of M. timonense and M. bouchedurhonense and identified subclusters within M. avium although no correlation with clinical manifestation was observed. We performed routine NGS to identify NTM from MGIT enriched shotgun metagenomic data. Phylogenetic analyses identified subtypes of M. avium, but in our set of isolates no correlation with clinical manifestation was found. However, this NGS workflow paves a way for more personalized health care in the future.

## INTRODUCTION

The genus Mycobacterium contains >200 different species, including the obligate pathogens Mycobacterium tuberculosis complex (MTBC) and M. leprae and the opportunistic pathogens classified as nontuberculous mycobacteria (NTM) (1, 2). NTM most frequently cause pulmonary disease (NTM-PD) in patients with preexisting pulmonary disease and/or immune deficiencies, but lymphadenitis, skin and soft tissue infections after inoculation, and disseminated disease in severely immunocompromised patients are also observed (2–4).

The clinical significance of isolation of NTM from nonsterile sites such as the airways differs strongly by species. In particular, the isolation of M. avium complex (MAC), M. abscessus, M. malmoense, M. xenopi, and M. kansasii often indicates true NTM pulmonary disease (4, 5). Antibiotic treatment regimens differ strongly by species (6). Therefore, correct species identification is crucial in the management of suspected mycobacterial disease.

Currently, molecular identification of NTM is performed using line probe assays (LiPAs), (multi)gene sequencing, or matrix-assisted laser desorption ionization–time of flight (MALDI-TOF) (7–12). Because these molecular methods target a small genomic region, exact identification of NTM to species level is not possible due to the high level of genetic similarity. Similarly, MALDI-TOF is able to differentiate only between a small subset of species and has reproducibility issues compared across multiple centers (11). We introduce a routine diagnostic next-generation sequencing (NGS) workflow that identifies mycobacteria to species and subspecies level from shotgun metagenomic data. In addition, we explored whether we are able to provide deeper insight into the NTM phylogeny and taxonomy. Together, this will contribute to more accurate identification and typing of NTM species, which in time may also allow correlating genotypes with clinical significance, disease manifestation, or even outcome.

## MATERIALS AND METHODS

### Clinical sample preparation.

New clinical samples were processed as previously described (13). In short, upon arrival nonsterile samples were pretreated with 2% *N*-acetyl-l-cysteine (NaLC)-NaOH for 15 min to decrease contamination. After pretreatment, phosphate buffer was added to a final volume of 50 mL. For materials obtained in sterile settings, pretreatment was skipped and phosphate buffer was immediately added to a final volume of 50 mL. Tubes were then centrifuged at 3,000 × *g* for 20 min at 4°C, all supernatant was discarded, 2 mL phosphate buffer was added, and the remaining sediment was vortexed briefly. An 0.5-mL amount of the sediment was then added to a liquid culture mycobacterial growth indicator tube (MGIT) containing 0.8 mL BD Bactec MGIT oleic acid-albumin-dextrose-catalase (OADC) growth supplement and PANTA (polymyxin B, amphotericin B, nalidixic acid, trimethoprim, and azlocillin) antibiotic supplement (BD Biosciences, Erembodegem, Belgium), and 0.25 mL was inoculated onto Lowenstein-Jensen (LJ) solid medium. Liquid culture samples were then incubated in the Bactec MGIT until they flagged positive. All MGIT-positive samples underwent further culture for strain storage including inoculation on blood agar to ensure a monoculture. Stored Mycobacterium species were cultured in Middlebrook 7H9 broth with 10% OADC supplement before pellet collection. Finally, solid medium cultures were swabbed and resuspended in glycerol broth. Samples were heat inactivated for DNA isolation.

### DNA isolation and library preparation.

DNA isolation was performed using InstaGene matrix (Bio-Rad, Veenendaal, the Netherlands) in combination with bead beating. Two hundred microliters InstaGene matrix was added to bacterial pellets, vortexed, and incubated with shaking at 500 rpm for 30 min at 56°C and then another 30 min at 99°C. Samples were then transferred to a tube containing <106-μm glass beads (Sigma-Aldrich, Saint Louis, MO, USA) and subjected to two rounds of bead beating at maximum speed in a MagNA Lyser (Roche, Woerden, the Netherlands). DNA integrity was measured on a TapeStation 2200 (Agilent, Santa Clara, CA, USA), and concentration was determined using a Qubit fluorometer (Thermo Fisher Scientific, Waltham, MA, USA). Library preparation was performed using the Illumina Nextera DNA Flex kit (Illumina, San Diego, CA, USA).

### Generation of a custom full-genome and *hsp65* mycobacterial species database.

We generated a curated database containing assembled genome sequences of 148 NTM type strains, including the most recent characterization of mycobacterial taxonomic relationships, performed by Tortoli et al. (14). Full genome sequences were retrieved from the NCBI genome database, and a next-generation sequencing–whole-genome (NGS-WG) database was constructed using a metagenomic classifier, Centrifuge (version 1.0.4beta) (15).

We also generated a database including the full-length *hsp65* gene from each strain. To this end, the ~1.6-kb fragment coding for the *hsp65* gene was retrieved from each of the 148 genomes and an NGS-*hsp65* typing database was constructed using KMA (version 1.3.2) (16).

All databases are made available via zenodo (17).

### Mycobacterial identification pipeline.

For processing the NGS data, we used our custom-developed pipeline MyCodentifier (version 1.0 beta) (https://jordycoolen.github.io/MyCodentifier/). In short, it is a Nextflow pipeline that cleans shotgun metagenomic NGS data and identifies the mycobacterial species up to sublineage level using the custom-built NGS-*hsp65* and NGS-WG databases. Results are reported if *hsp65* identity score and coverage are ≥70%; elsewise, they are reported as failed.

### MyCodentifier pipeline validation.

**FIG 1:**
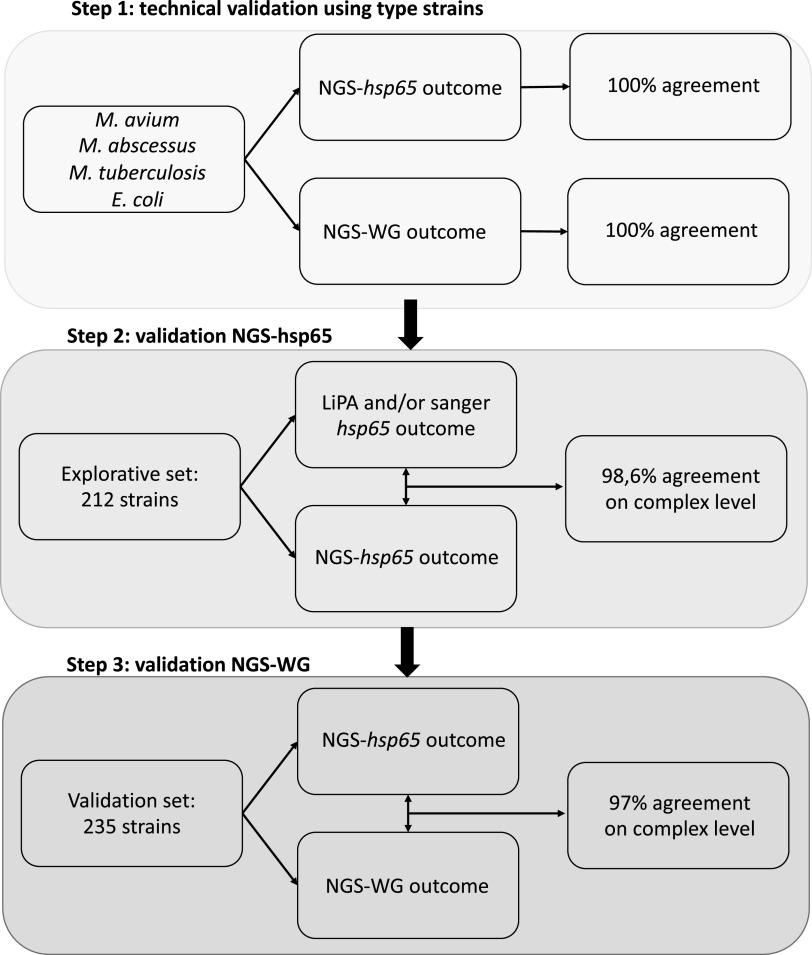
MyCodentifier pipeline validation flowchart.

Our sample preparation and MyCodentifier pipeline were validated in three separate rounds shown in Fig. 1. First, a technical validation was performed using the Mycobacterium avium ATCC 700898, Mycobacterium tuberculosis H37Rv, Mycobacterium abscessus CIP 104536, and Escherichia coli ATCC 25922 type strains. Subsequently, we validated the use of the NGS-*hsp65* typing database in the MyCodentifier pipeline using an explorative set of 219 samples that were included between March 2019 and August 2020, shown in Table 1. Species identification was compared to that obtained using the Inno-LiPA Mycobacteria v2 line probe assay (performed according to manufacturer’s instructions); when a result of “*M. genus*” (unknown mycobacterial species) was obtained, the analysis was supplemented with partial *hsp65* gene sequencing (Telenti fragment [18]) to reach species-level identifications. Finally, we validated the use of the NGS-WG identification database for species determination using a validation set of 235 strains sequenced between January and June 2021, shown in Table 2. Samples were included if the data contained more than 1 million reads, nonmycobacterial DNA contamination was less than 30%, average sequence depth was ≥30× sequence reads for *hsp65*, and identity and coverage were ≥80%. To validate the whole-genome results, we compared the outcome to that of the previously validated *hsp65* database.

**TABLE 1:** NGS-*hsp65* typing database results compared to Inno-LiPA Mycobacteria v2 line probe and partial *hsp65*

NGS-*hsp65*			Inno-LiPA Mycobacteria v2 line probe												
		Group	M. abscessus	M. avium complex				*M. celatum* complex	M. chelonae	M. fortuitum*-peregrinum* complex	M. gordonae	M. haemophilum	M. kansasii		
		Species	M. abscessus	M. avium	M. avium complex	*M. chimaera*	M. intracellulare	*M. celatum*	M. chelonae	M. fortuitum*-peregrinum* complex	M. gordonae	M. haemophilum	M. kansasii	M. kansasii group I	M. kansasii group II
Group	Species														
M. abscessus	M. abscessus subsp. *abscessus*		17												
	M. abscessus subsp. *bolletii*		3												
	M. abscessus subsp. *massiliense*		5												
M. avium	M. avium			16											
	M. bouchedurhonense			30											
	M. timonense			17					1*^a^						
M. avium complex (MAC)	*M. chimaera*					30									
	M. colombiense				1										
	M. intracellulare					1	17								
	M. marseillense				2		1								
	*M. yongonense*				1	1	2								
*M. celatum* group	*M. celatum*							3							
M. chelonae	M. chelonae								12						
*M. elephantis*	*M. elephantis*														
M. fortuitum*-peregrinum* group	M. fortuitum						1*			10					
	*M. houstonense*									2					
	*M. peregrinum*									2					
	*M. senegalense*									1					
M. gordonae	M. gordonae										8				
M. haemophilum	M. haemophilum											3			
M. kansasii group	M. kansasii												1	7	
	*M. persicum*														2
M. tuberculosis	M. tuberculosis						1*							

			**Inno-LiPA Mycobacteria v2 line-probe**				**Partial *hsp65***								
		**Group**	**M. marinum** **group**	**M. simiae complex**	**M. tuberculosis complex**	**M. xenopi complex**	**“*M. genus*”**								
**NGS-hsp65**		**Species**	**M. marinum/M. ulcerans**	**M. simiae**	**M. tuberculosis complex**	**M. xenopi**	***M. elephantis***	**M. fortuitum/M. houstonense/M. farcinogenes**	***M. kumamotonense***	**M. mucogenicum**	***M. phlei***	***M. stomatepiae***			
**Group**	**Species**														
*M. celatum* group	*M. celatum*														
M. chelonae	M. chelonae														
*M. elephantis*	*M. elephantis*						1								
M. fortuitum*-peregrinum* group	M. fortuitum														
	*M. houstonense*							1							
	*M. peregrinum*														
	*M. senegalense*														
M. gordonae	M. gordonae														
M. haemophilum	M. haemophilum														
M. kansasii group	M. kansasii														
	*M. persicum*														
M. marinum group	M. marinum		2												
M. mucogenicum	M. mucogenicum									1					
*M. phlei*	*M. phlei*										1				
M. simiae complex	*M. lentiflavum*											1			
	M. simiae			3											
*M. terrae* complex	*M. kumamotonense*								1						
M. tuberculosis	M. tuberculosis				2										
M. xenopi group	M. xenopi					1									

a *, discrepancy in result.

**TABLE 2:** NGS-*hsp65* typing database results compared to NGS-WGS typing^*a*^

NGS-*hsp65*			NGS-WGS														
			M. abscessus *chelonae* complex				M. avium complex (MAC)									*M. celatum* group	
			M. abscessus subsp. *abscessus*	M. abscessus subsp. *bolletii*	M. abscessus subsp. *massiliense*	M. chelonae	M. avium	M. bouchedurhonense	*M. chimaera*	M. colombiense	M. intracellulare	M. marseillense	M. paraintracellulare	M. timonense	*M. yongonense*	*M. shimoidei*	
M. abscessus *chelonae* complex	M. abscessus subsp. *abscessus*		11														
	M. abscessus subsp. *bolletii*			2													
	M. abscessus subsp. *massiliens*				6												
	M. chelonae					23											
M. avium complex	M. avium						18										
	M. bouchedurhonense						11^	7			1^			8^			
	*M. chimaera*								26		1^		1^				
	M. colombiense									2							
	M. intracellulare										7		1^		2^		
	M. marseillense											2					
	M. timonense													4			
	*M. yongonense*								1^						3		
*M. celatum* group	*M. shimoidei*															1	
M. fortuitum*-smegmatis* group	*M. boenickei*																
	M. fortuitum																
	*M. peregrinum*																
	M. porcinum																
	*M. septicum*																
M. marinum group~	M. marinum																
	M. ulcerans																
*M. terrae* complex	*M. arupense*																
	*M. heraklionense*																
	*M. kumamotonense*																
M. xenopi group	*M. noviomagense*																
	M. xenopi																
Pathogen group	M. gordonae																
	M. kansasii																
	M. tuberculosis																
NA	*M. interjectum*															
	*M. malmoense*															
	M. mucogenicum					1*								

		**NGS-WGS**															
		***M. celatum* group**	**M. fortuitum*-smegmatis* group**				**M. marinum group~**	***M. terrae* complex**			**M. xenopi group**	**Pathogen group**				**NA**	
**NGS-*hsp65***		***M. shimoidei***	**M. fortuitum**	***M. peregrinum***	**M. porcinum**	***M. septicum***	***M. pseudoshottsii***	***M. arupense***	***M. heraklionense***	***M. kumamotonense***	***M. noviomagense***	**M. xenopi**	**M. gordonae**	**M. kansasii**	**M. tuberculosis**	***M. interjectum***	***M. malmoense***
*M. celatum* group	*M. shimoidei*	1														
M. fortuitum*-smegmatis* group	*M. boenickei*				1^											
	M. fortuitum		8														
	*M. peregrinum*			1													
	M. porcinum				1												
	*M. septicum*					3											
M. marinum group~	M. marinum						4#									
	M. ulcerans						1#										
*M. terrae* complex	*M. arupense*							1									
	*M. heraklionense*								1								
	*M. kumamotonense*									1							
M. xenopi group	*M. noviomagense*										2						
	M. xenopi											4					
Pathogen group	M. gordonae												16				
	M. kansasii													9			
	M. tuberculosis														41		
NA	*M. interjectum*															1
	*M. malmoense*															1	
	M. mucogenicum																

a *, discrepancy in result; ~, *M. marinum* group is sometimes referred to as *M. marinum-ulcerans* group; ^, different subspecies in MAC; #, different subspecies in *M. marinum* group; NA, not applicable.

### Database update.

The NGS-WG typing database was later expanded by adding additional reference genomes to improve NTM subspecies identification, one M. marinum and three M. avium subspecies retrieved from the NCBI genome database (see Table S1 in the supplemental material).

### Bioinformatic analysis of phylogeny.

A detailed description of bioinformatic analysis and visualization can be found in the Supplementary Methods in the supplemental material. In short, kSNP3 was used to calculate 0.5 majority single nucleotide polymorphism (SNP) parsimony trees of the M. marinum-M. ulcerans group, M. avium complex, and M. abscessus using sequence reads obtained from MyCodentifier together with multiple reference genomes (Table S1) (19–22).

## RESULTS

### Validation phase. (i) Technical validation.

All M. tuberculosis H37Rv *and* M. abscessus ATCC 19977 type strains were correctly identified; all M. avium ATCC 700898 type strains were identified as M. bouchedurhonense, a member of the Mycobacterium avium complex (MAC) almost indistinguishable from M. avium (23). All negative controls included in the technical validation failed species identification, and all E. coli samples were flagged as contaminated.

### (ii) Clinical validation of the NGS-*hsp65* typing database.

Two hundred nineteen isolates with an NGS-*hsp65* typing result and a LiPA result were included for clinical validation. Seven samples had previously been identified as mixed infections during culture or based on their LiPA result and were removed from the validation set. Six samples did not have a specific LiPA typing (“*M. genus*”) and were typed using partial *hsp65* Sanger sequencing. Of the remaining 212 isolates, 98.6% (*n* = 209) had the same outcome on the complex level (Table 1).

### Discrepancy analysis.

Within certain species or complexes (M. abscessus, M. kansasii, MAC, and M. fortuitum*-*M. peregrinum complex), the NGS-*hsp65* typing was able to identify (sub)species within the complex as reported by the LiPA (Table 1). For example, within MAC we identified eight species of MAC that are not present on the LiPA and had been previously identified as MAC or one of the species included in the LiPA (M. avium, M. intracellulare, and M. chimaera). One NGS-*hsp65* (M. intracellulare) species identification differed from the LiPA identification (*M. chimaera*). One M. simiae complex isolate identified by partial *hsp65* Sanger sequencing as M. stomatepiae (not included in the Tortoli et al. database [14]) was identified as the closely related M. lentiflavum using NGS-*hsp65*.

Discrepancy analysis, consisting of reviewing all steps from culture to analysis, of the three strains not explained by differences in databases was performed. One case was found to be due to a pipetting error (M. tuberculosis-M. intracellulare). Additional errors (M. fortuitum-M. intracellulare and M. timonense-M. chelonae) were untraceable but are likely due to similar human error.

### Clinical diagnostics using NGS whole-genome typing.

As we aim to use NGS-WG in clinical practice, the NGS-*hsp65* outcome was compared to NGS-WG for 235 strains (Table 2). On the complex level, our findings show a 97.0% agreement between NGS-*hsp65* and NGS-WG identification. If we include strains within the closely related M. marinum*-*M. ulcerans*-*M. pseudoshottsii cluster, it increases to 99.1%. One strain differed between NGS-WG (M. chelonae) and NGS-*hsp65* (M. mucogenicum) due to a mixed infection. The second discrepancy between NGS-WG (M. arupense) and NGS-*hsp65* (M. gordonae) was due to the abundance calculation incorporated in the centrifuge typing tool, in which small genomes, like *M. arupense*, result in high abundance percentages despite lower numbers of reads. Upon human curation the isolate was identified as M. gordonae. On species level an accordance of 85.2% is found; the 14.8% observed discrepancies consist mainly of MAC species (11.4%) and M. marinum*-M. pseudoshottsii* (2.1%). One discrepancy (0.4%) is found between M. porcinum and M. boenickei, closely related strains within the M. fortuitum*-smegmatis* group. For M. abscessus, identification up to subspecies level was achieved for all isolates (see Fig. S1 in the supplemental material).

### M. marinum and MAC phylogeny analysis.

**FIG 2:**
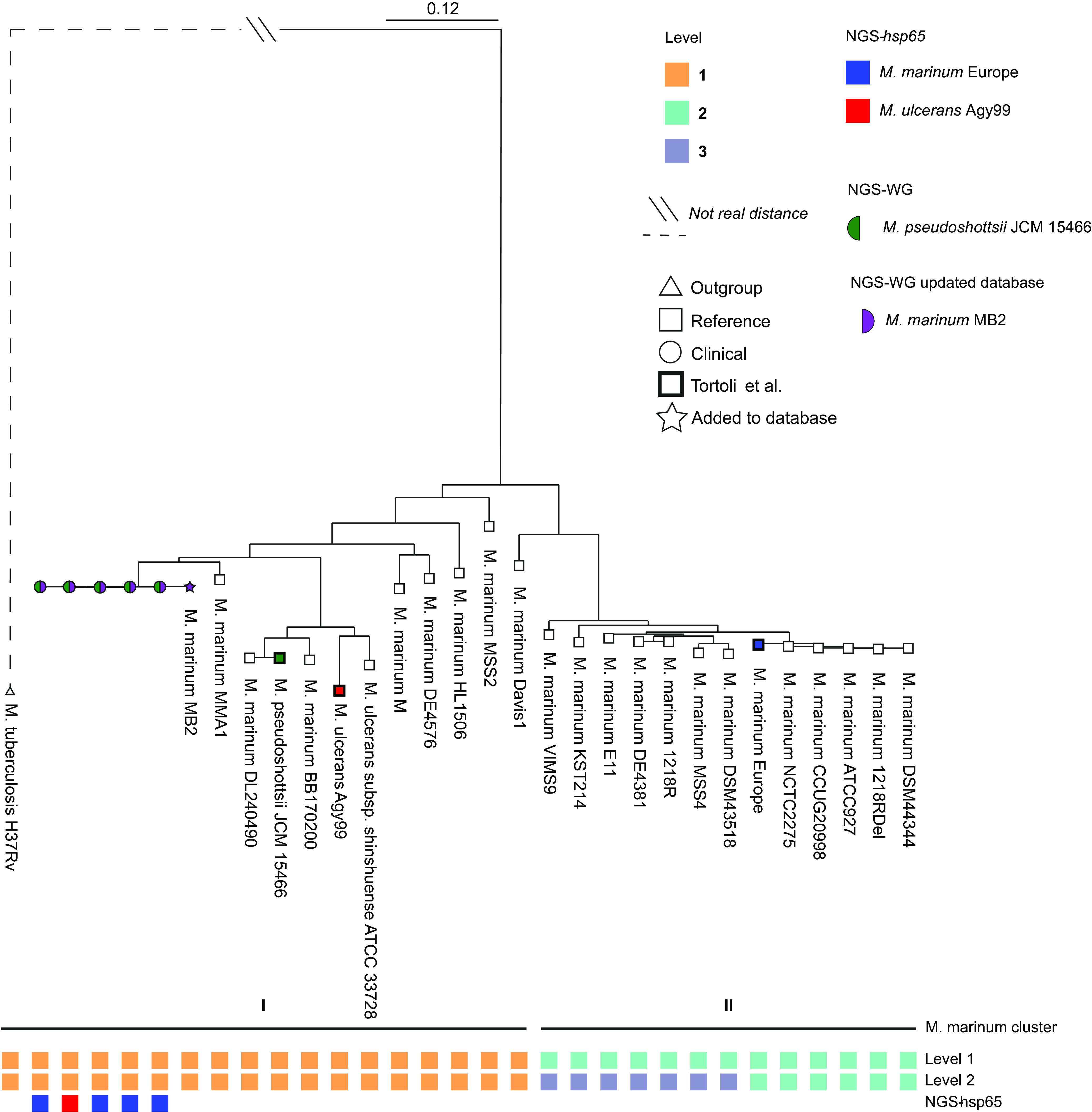
M. marinum group phylogeny, illustrating the clinical samples being similar and being closely related to M. marinum MB2. The discrepancy of NGS-*hsp65* and NGS-WG typing can be clarified by the absence of reference from cluster 1 in the Tortoli et al. database. Levels of fastBAPS identify on level 1 two clear distinct clusters of M. marinum. On level 2 the second cluster is divided into two separate clusters.

The discrepancies between the NGS-*hsp65* and NGS-WG typing within the M. marinum group and MAC were studied further. We performed phylogenetic analysis of all five clinical strains belonging to the M. marinum group together with 25 reference genomes retrieved from NCBI (Fig. 2; included strains are shown in Table S1) and M. tuberculosis H37Rv as outgroup. We show that within M. marinum two clusters of strains are identified. Because the strain database derived from the work of Tortoli et al. (14) includes only two strains from cluster I (*M. pseudoshottsii* JCM15466 and M. ulcerans Agy99) and one M. marinum strain from cluster II (M. marinum Europe), MyCodentifier identified all clinical M. marinum strains as *M. pseudoshottsii*; however, they should have been identified as a cluster I M. marinum (Fig. 2). After adding M. marinum MB2, belonging to cluster I, to the database, all strains were correctly typed.

**FIG 3:**
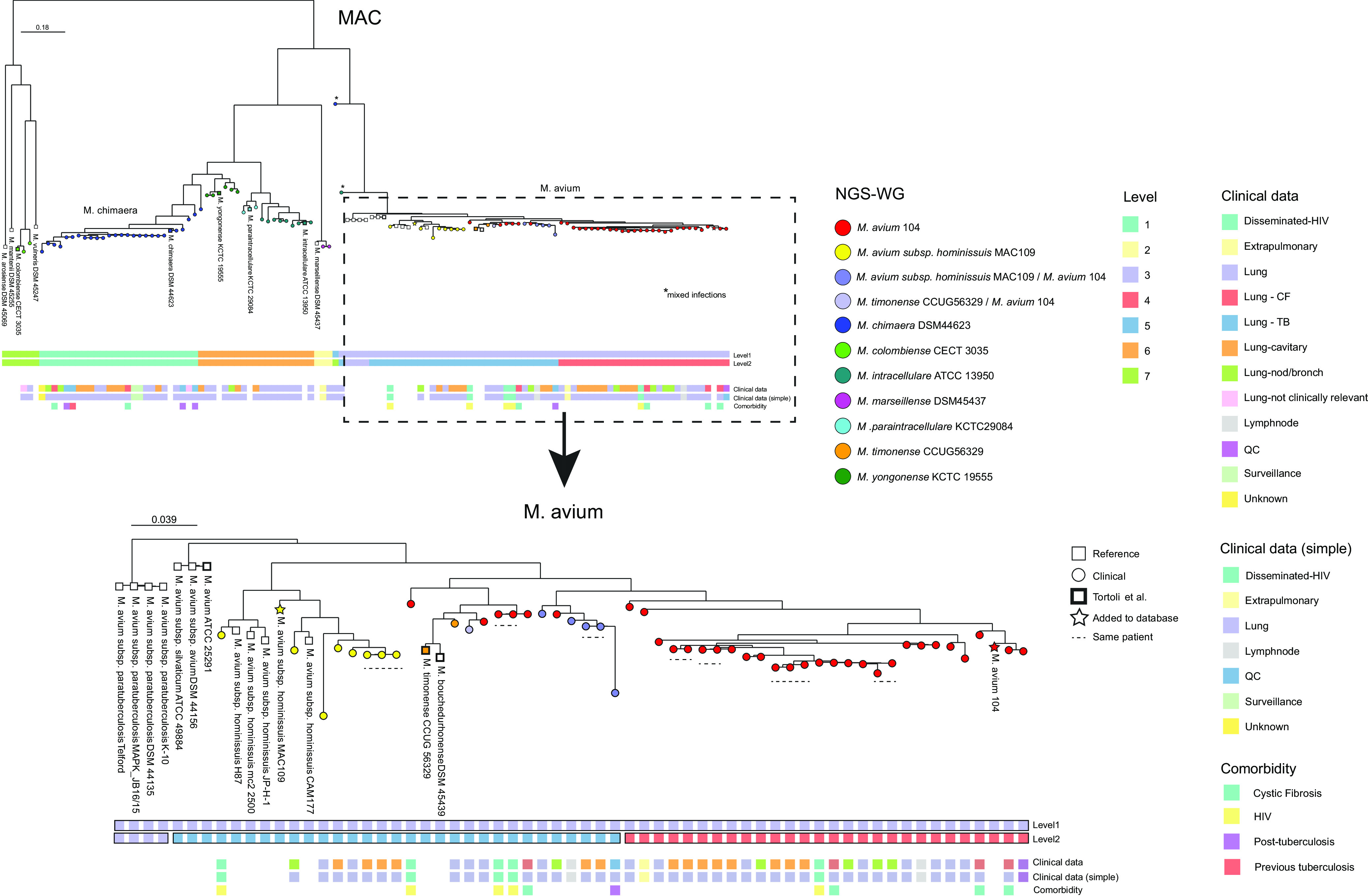
M. avium complex phylogeny and M. avium in more detail. The phylogeny illustrates that the NGS-WG typing is perfectly able to identify the species of MAC. However, it has some trouble in determining the M. avium subspecies. This is mainly because the phylogeny shows multiple small subclusters of M. avium. fastBAPS cluster 3 (on level 2) shows distinct Mycobacterium avium subsp. *paratuberculosis* cluster. fastBAPS cluster 4 (on level 2) shows a distinct M. avium 104-like cluster. CF, cystic fibrosis; TB, tuberculosis; QC, quality control; nod/bronch, nodule/bronchiole; HIV, human immunodeficiency viruses.

We then performed phylogenetic analysis of all MAC isolates (*n* = 95) and 24 reference sequences from NCBI (Table S1). Phylogenetic analysis shows that MAC contains multiple well-defined clusters, largely related to existing (sub)species. Overall, the phylogenetic tree supports the findings from the NGS-WG and not the NGS-*hsp65* typing (Fig. 3). One exception is formed by the M. avium, M. bouchedurhonense, and M. timonense cluster, species which do not cluster based on NGS-WG typing. Two strains that did not cluster were identified as mixed infections during manual curation and confirmed to be a mixed infection by splitting colonies from either the LJ medium or the blood agar during classical culture workup and identified using the same NGS workflow. After updating the database with additional M. avium strains and rerunning the analysis (Table S2), four clusters of clinical isolates are observed (Fig. 3). One cluster was comprised of strains closely related to Mycobacterium avium subsp. *hominissuis* 109. A second cluster was formed by strains closely related to Mycobacterium avium subsp. *avium* 104. The remaining two clusters lay between these two defined groups and were comprised of either strains clearly typed as M. avium subsp. *avium* 104 or M. timonense or strains that were related to a combination of M. avium 104 and M. timonense or a combination of M. avium subsp. *hominissuis* 109 and M. avium 104. Finally, the site of disease manifestation (pulmonary, extrapulmonary, disseminated, or lymph nodes) and the presence of other relevant diseases (cystic fibrosis, HIV, or current tuberculosis) were recorded for all M. avium isolates, and no correlation was seen with specific clusters. Our validation set contained a number of strains sequenced sequentially from the same patient (Fig. 3). Duplicate cultures cluster together in all but one case, likely explained by reinfection of a patient with a novel strain.

We calculated the phylogenetic tree of all M. abscessus subspecies and illustrate a clear separation of subspecies (Fig. S1; included strains shown in Table S1).

## DISCUSSION

**FIG 4:**
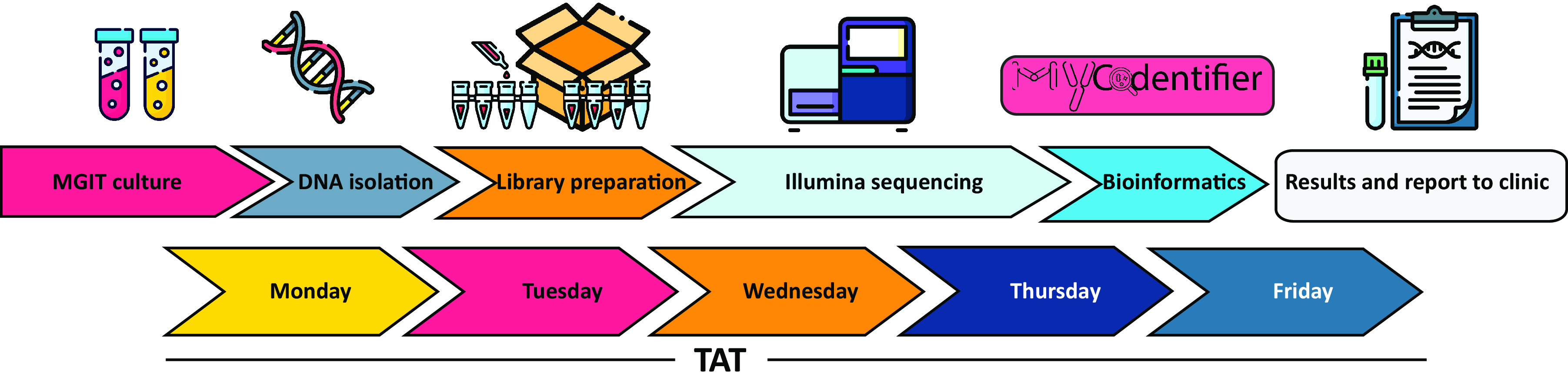
Flowchart of routine use of MGIT enriched shotgun metagenomics for identification of nontuberculous mycobacteria. All the steps are shown in the flowchart and projected onto our weekly planning. As this workflow is repeated weekly. the maximum time from positive MGIT to DNA isolation is 7 days, meaning the TAT is 4 to 11 days. Icons are obtained from flaticon and are free to use.

Implementation of whole-genome-based NGS identification of mycobacteria using the MyCodentifier in our clinic proved accurate and efficient in routine practice. Previous mycobacterial identification methods have significant drawbacks, the most important being the limited number of included strains in commercial and validated methods such as LiPA and MALDI-TOF. Although partial 16S rRNA, *rpoB*, or *hsp65* sequencing allowed for identification of most species, it depends on the quality of sequences uploaded to noncurated online databases. The implementation of NGS-WG allows for highly specific and reproducible identifications, including identification of mixed infections, and it allows typing to subspecies level and beyond. In our current setting, the turnaround time (TAT) of WGS will not significantly delay the diagnosis in the context of a chronic infection with a slow-growing bacterium but will slightly delay species identification. With our current NGS-WG method, the TAT from positive MGIT to species determination is between 4 and 11 days, depending on the day the MGIT flags positive (Fig. 4). With our previous methodology (LiPA supplemented with Sanger sequencing), the TAT from positive MGIT for the LiPA was between 2 and 5 days, and if more in-depth typing was necessary using Sanger sequencing, it was between 7 and 8 days. While the implementation of NGS-WG for NTM diagnostics may now be most useful in specialized centers, as the incidence of NTM and the use of NGS in routine microbiology laboratories increases while the accompanying cost decreases, the benefits of this technique in the place of MALDI-TOF or LiPA will likely warrant use in broader settings.

Within M. abscessus, our NGS-WG pipeline allowed identifications up to subspecies level (see Fig. S1 in the supplemental material); this is important because the incidence of macrolide susceptibility differs between M. abscessus subspecies and, as a result, so does the chance of successful antibiotic treatment (6). It may be relevant to add representative genomes of the recently identified dominant circulating clones to the database, for rapid identification of these clinically and epidemiologically important clones (24).

We completed phylogenetic analysis to study the M. marinum clinical isolates. Our findings confirm that the M. marinum group consists of two clusters (20). One cluster includes the mycolactone-producing strains M. ulcerans and *M. pseudoshottsii* (cluster I), and the other includes M. marinum Europe (cluster II). Because our clinical M. marinum strains belonged to cluster I, they were initially identified as *M. pseudoshottsii*, the nearest ancestor present in the Tortoli et al. phylogeny (14). However, in our phylogenetic analysis our clinically isolated M. marinum strains clustered with M. marinum strains known not to carry the plasmid containing the mycolactone-producing gene and did not clinically present Buruli ulcers and are therefore not mycolactone-producing mycobacteria (20). The addition of the cluster I M. marinum MB2 strain to the database confirmed that the clinical strains were correctly identified as being part of cluster I (Fig. 2).

With phylogenetic analysis within the MAC we show that differentiation between most (sub)species is possible using typing on NGS-WG level and on NGS-*hsp65*. Nevertheless, subspecies identification using NGS-*hsp65* for M. avium, M. avium subspecies, M. bouchedurhonense, and M. timonense subgroups proved difficult. Previously reported findings support that it is difficult to discriminate between M. avium subspecies and that M. bouchedurhonense and M. timonense are minimally different from M. avium and as such do not merit a separate species status (14, 23). We therefore report all M. bouchedurhonense and M. timonense as M. avium to the clinic (Table S2). Interestingly, we see four distinct clusters within the sequenced M. avium strains. Previous studies have proposed that these clusters may represent strains of M. avium responsible for different disease manifestations or affecting different patient groups (25, 26). We found no such associations, though the data set was likely too small to investigate possible correlations with specific M. avium clusters. Moving forward, our collection of NGS-WG-identified isolates will increase in numbers and will help to determine whether a disease manifestation, disease severity, or treatment outcome is correlated with genotype.

Although automated analysis is convenient, it remains important that results are manually curated prior to reporting to the clinic. In our clinic manual curation is performed by a multidisciplinary team consisting of at least a specialized bioinformatician and medical microbiologist and sometimes a laboratory specialist when necessary. The need for manual curation is exemplified by the incorrect identification of an M. gordonae isolate as *M. arupense*, resulting from the black-and-white calculation methods underlying the bioinformatic pipeline. Human curation is also necessary to identify mixed infections based on the presence of reads mapping to multiple species. The current report not only highlights the most prevalent species but also includes a table with the 10 most dominant species, thereby allowing us to manually identify the presence of multiple species in one sample. We believe that this, together with laboratory sample exchange, is likely the reason for the small percentage of incorrectly typed strains relative to the LiPA. Furthermore, quality control of the WGS-NG workflow and pipeline is performed regularly by the inclusion of known positive and negative samples and monitored by the multidisciplinary team. In addition, although our current database is effective in identifying the majority of NTM isolates to subspecies level, 10 strains could not be identified. This is likely due to the limitations of our database. We included all strains in the database of Tortoli et al., because this is currently the most extensively described, curated database (14). However, it does not include all described strains (1). In addition, many species of NTM likely remain uncharacterized, and unidentifiable isolates may thus represent novel species. Moving forward, we aim to characterize novel strains, preferably using long-read sequencing such as Oxford Nanopore Technologies or PacBio sequencing to obtain high-quality genomes (27, 28), and update the database periodically to include them.

In contrast to NGS studies of M. tuberculosis complex organisms (29), we have not yet validated NGS-WG to detect antibiotic resistance-associated mutations. Mechanisms of antibiotic resistance in NTM are incompletely understood. Although detection of known resistance-conferring mutations in 16S (for amikacin) or 23S (for macrolides) rRNA genes can predict resistance (30), prediction of susceptibility in the absence of known mutations is difficult due to more complex mechanisms of resistance in, for example, M. abscessus (31), and in less-well-studied NTM species.

In conclusion, we present a novel methodology for identification of clinically isolated mycobacteria using NGS-WG that has multiple benefits over conventional typing methods. First, this method allows for more in-depth identifications to species or even subspecies level, often relevant for treatment regimens or understanding virulence. Second, fewer additional tests are needed to perform detailed identification and typing. The detection of antibiotic resistance in NTM, the presence of plasmids, and the associations between genotypes and clinical manifestations or virulence are important topics for future studies.
