# Supplementary material for: MGIT Enriched Shotgun Metagenomics for Routine Identification of Nontuberculous Mycobacteria: a Route to Personalized Health Care
Source: J Clin Microbiol. 2023 Feb 22;61(3):e01318-22. doi: 10.1128/jcm.01318-22 (PMC10035320; doi:10.1128/jcm.01318-22)
Supplement: Supplemental file 1 — Supplemental material. Download jcm.01318-22-s0001.pdf, PDF file, 0.4 MB [file jcm.01318-22-s0001.pdf]

Supplementary material

Supplementary methods

Bioinformatic analysis of phylogeny

Cleaned sequence reads were obtained from MyCodentifier and converted to fasta files using seqtk (version 1.3-r106) [1]. kSNP3 (version 3.1) was used to calculate 0.5 majority SNP parsimony trees of the *M. marinum-M. ulcerans* group, *M. avium complex* and *M. abscessus* [2-5]. For all analysis multiple reference genomes were included (supplementary table 1). Clusters levels were computed using fastBAPS (version 1.0.6) [6]. Visualization of the results was performed using next Microreact (version 0.1.0). SNP distances were calculated on the 0.5 majority SNP alignment (containing only SNPs present in over 50% of genomes) using snp-dists (version 0.7.0) and visualized using R (version 4.0.5) and ggplot2 (version 3.3.5) [7-9].

References

1. Shen, W., et al., *SeqKit: A Cross-Platform and Ultrafast Toolkit for FASTA/Q File Manipulation.* PLoS One, 2016. **11**(10): p. e0163962.
2. Gardner, S.N., T. Slezak, and B.G. Hall, *kSNP3.0: SNP detection and phylogenetic analysis of genomes without genome alignment or reference genome.* Bioinformatics, 2015. **31**(17): p. 2877-8.
3. Das, S., et al., *Extensive genomic diversity among Mycobacterium marinum strains revealed by whole genome sequencing.* Sci Rep, 2018. **8**(1): p. 12040.
4. Rindi, L. and C. Garzelli, *Genetic diversity and phylogeny of Mycobacterium avium.* Infect Genet Evol, 2014. **21**: p. 375-83.
5. Lee, M.R., et al., *Mycobacterium abscessus Complex Infections in Humans.* Emerg Infect Dis, 2015. **21**(9): p. 1638-46.
6. Tonkin-Hill, G., et al., *Fast hierarchical Bayesian analysis of population structure.* Nucleic Acids Res, 2019. **47**(11): p. 5539-5549.
7. Argimón, S., et al., *Microreact: visualizing and sharing data for genomic epidemiology and phylogeography.* Microb Genom, 2016. **2**(11): p. e000093.
8. Seemann, T., *Pairwise SNP distance matrix from a FASTA sequence alignment.* 2018.
9. Wickham, H., *ggplot2: Elegant Graphics for Data Analysis.* Springer-Verlag New York. isbn: 978-3-319-24277-4, 2016.

Supplementary figures and tables

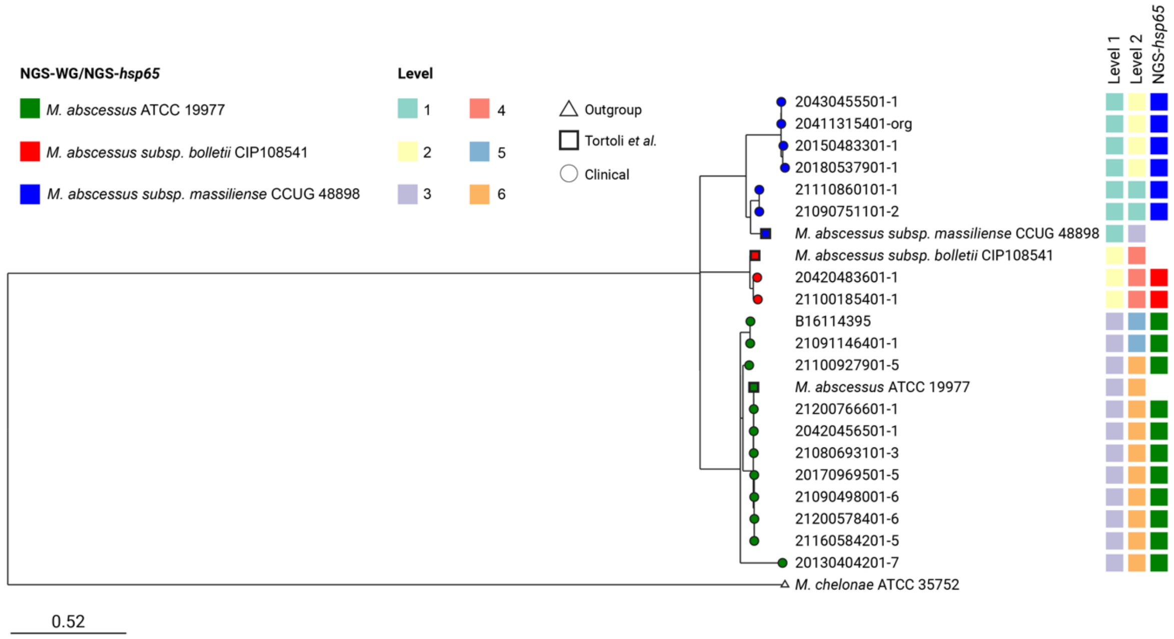

Supplementary Figure 1: *M. abscessus* phylogeny.

Clinical samples of *M. abscessus* and there NGS-*hsp65* and NGS-WG typing results showing 100% correct identification to subspecies level. fastBAPS on level 1 also identifies the three subspecies of *M. abscessus*. *M. chelonae* ATCC 35752 was used as outgroup.

Supplementary table 1. Reference genomes used in phylogenetic analysis.

Mycobacterium marinum - ulcerans - pseudoshottsi			
**Strain**	**Tortoli *et al.***	**Added**	**Accession**
Mycobacterium marinum E11	No		GCF_002911575.1
Mycobacterium marinum Europe	Yes		GCF_000419315.1
Mycobacterium marinum M	No		GCF_000018345.1
Mycobacterium marinum MB2	No	Yes	GCF_000419335.1
Mycobacterium marinum strain 1218R	No		GCF_003391415.1
Mycobacterium marinum strain 1218RDel	No		GCF_002911515.1
Mycobacterium marinum strain ATCC 927	No		GCF_003609695.1
Mycobacterium marinum strain BB170200	No		GCF_003431765.1
Mycobacterium marinum strain CCUG20998	No		GCF_003391395.1
Mycobacterium marinum strain Davis1	No		GCF_003431665.1
Mycobacterium marinum strain DE4381	No		GCF_003431585.1
Mycobacterium marinum strain DE4576	No		GCF_003431805.1
Mycobacterium marinum strain DL240490	No		GCF_003431775.1
Mycobacterium marinum strain DSM 43518	No		GCF_003431565.1
Mycobacterium marinum strain DSM 44344	No		GCF_003431645.1
Mycobacterium marinum strain HL1506	No		GCF_002249865.1
Mycobacterium marinum strain KST214	No		GCF_003431675.1
Mycobacterium marinum strain MMA1	No		GCF_016745295.1
Mycobacterium marinum strain MSS2	No		GCF_003431735.1
Mycobacterium marinum strain MSS4	No		GCF_003431725.1
Mycobacterium marinum strain NCTC2275	No		GCF_003431655.1
Mycobacterium marinum strain VIMS9	No		GCF_003431575.1
Mycobacterium pseudoshottsii JCM 15466	Yes		GCF_001515365.1
Mycobacterium tuberculosis H37Rv	Yes		GCF_000195955.2
Mycobacterium ulcerans Agy99	Yes		GCF_000013925.1
Mycobacterium ulcerans subsp. shinshuense ATCC 33728	No		GCF_002355775.1
***Mycobacterium avium* complex (MAC)**			
**Strain**	**Tortoli *et al.***	**Added**	**Accession**
Mycobacterium arosiense DSM 45069	Yes		GCF_002086125.1
Mycobacterium avium 104	No	Yes	GCF_000014985.1

Mycobacterium avium ATCC 25291	Yes		GCF_000174035.1
Mycobacterium avium subsp. avium DSM 44156	No		GCF_009741445.1
Mycobacterium avium subsp. hominissuis CAM177	No		GCF_002304065.1
Mycobacterium avium subsp. hominissuis H87	No		GCF_001936215.1
Mycobacterium avium subsp. hominissuis JP-H-1	No		GCF_009002535.1
Mycobacterium avium subsp. hominissuis MAC109	No	Yes	GCF_003408535.1
Mycobacterium avium subsp. hominissuis mc2 2500	No		GCF_004345205.2
Mycobacterium avium subsp. paratuberculosis DSM 44135	No	Yes	GCF_013357385.1
Mycobacterium avium subsp. paratuberculosis K-10	No		GCF_000007865.1
Mycobacterium avium subsp. paratuberculosis MAPK_JB16/15	No		GCF_003815815.1
Mycobacterium avium subsp. paratuberculosis Telford	No		GCF_003957335.1
Mycobacterium avium subsp. silvaticum ATCC 49884	No		GCF_000504975.1
Mycobacterium bouchedurhonense DSM 45439	Yes		GCF_002086165.1
Mycobacterium chimaera DSM 44623	Yes		GCF_002101575.1
Mycobacterium colombiense CECT 3035	Yes		GCF_000222105.2
Mycobacterium intracellulare ATCC 13950	Yes		GCF_000277125.1
Mycobacterium intracellulare yongonense KCTC 19555	Yes		GCF_000418535.1
Mycobacterium mantenii DSM 45255	Yes		GCF_002086335.1
Mycobacterium marseillense DSM 45437	Yes		GCF_002086345.1
Mycobacterium paraintracellulare KCTC 29084	No		GCF_002104735.1
Mycobacterium timonense CCUG 56329	Yes		GCF_002086775.1
Mycobacterium vulneris DSM 45247	Yes		GCF_002104765.1
Mycobacterium abscessus - chelonae			
**Strain**	**Tortoli *et al.***	**Added**	**Accession**
Mycobacterium abscessus ATCC 19977	Yes		GCF_000069185.1
Mycobacterium abscessus subsp. bolletii CIP108541	Yes		GCF_002086525.1
Mycobacterium abscessus subsp. massiliense CCUG 48898	Yes		GCF_002086375.1
Mycobacterium chelonae ATCC 35752	Yes		GCF_002102055.1

Supplementary table 2. NGS-WG typing results before (vertical) and after (horizontal) MAC database update.

		Updated database NGS-WG					
NGS-WG		M. avium 104	M. avium 104 / M. timonense CCUG56329	M. avium hominissuis MAC109	M. avium hominissuis MAC109 / M. avium 104	M. timonense CCUG56329	Total
	M. avium ATCC 25291	28		1			29
	M. bouchedurhonense DSM45439	1		2	4		7
	M. timonense CCUG56329	4	1	5	1	1	12
	Total	33	1	8	5	1	48
